# 151. Association Between Outpatient Antibiotic Prescribing, Antimicrobial Resistance, and Initial Presentation to Inpatient Setting for Urinary Tract Infections Among Older Adults in New York State

**DOI:** 10.1093/ofid/ofab466.353

**Published:** 2021-12-04

**Authors:** Marissa N Grillo, Joshua Barlow, Joseph Carreno

**Affiliations:** Albany College of Pharmacy and Health Sciences, Secaucus, New Jersey

## Abstract

**Background:**

Antibiotic prescribing (AP) and resistance (AR) may influence severity of illness in urinary tract infection (UTI). Limited data exist assessing the relationship between county-level AP and AR on initial presentation to hospital for UTI. This study evaluated the association between county-level AP and AR on UTI severity of illness among hospitalized patients in New York State.

**Methods:**

Retrospective, cross-sectional analysis, combining data from New York State Statewide Planning and Research Cooperative System (SPARCS) and previously published data on countywide antimicrobial resistance and antimicrobial prescribing. Inclusion criteria: female patients admitted to a New York inpatient setting in 2017, UTI (CCS 159), Medicare insurance. Exclusion criteria: missing countywide prescribing or resistance. All-patient refined (APR) clinical severity ≥ 3 was the primary outcome. Counties were classified as prescribing above or below the median prescribing proportion, and above or below the median prevalence of E. coli resistance for TMP-SMX and NTF. Countywide prescribing practices, antimicrobial resistance, patient factors, and location factors were evaluated for association with APR clinical severity ≥ 3 using chi-squared and logistic regression.

**Results:**

8,024 patients met study criteria. Baseline characteristics are presented in Table 1. 3,597 (44.8%) had an APR severity of ≥ 3. Factors associated with APR severity ≥ 3 include age group (P < 0.001), ethnicity (P = 0.013), hospital county (P < 0.001), first line prescribing ≥ 45.4% (P = 0.049), E. coli TMP-SMX resistance ≥ 29.0% (P < 0.001) via chi-squared test. In the logistic regression analysis counties with higher first line prescribing was associated with decreased odds for severe infection (aOR: 0.83 [0.72 – 0.97]). Additional factors associated with severe infection are presented in Table 2.

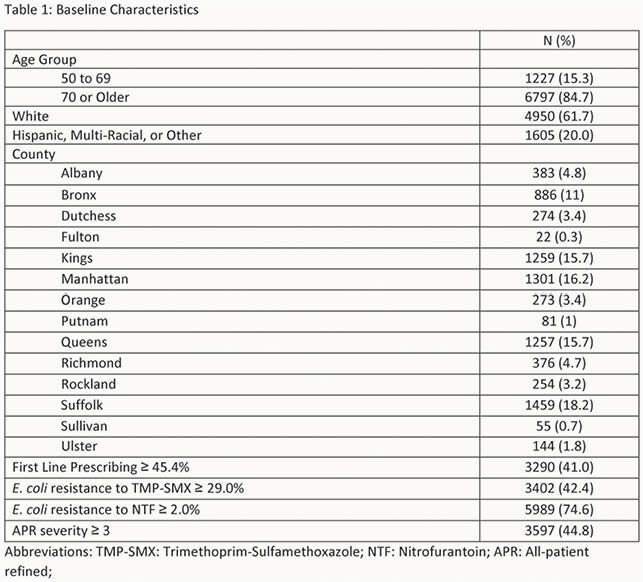

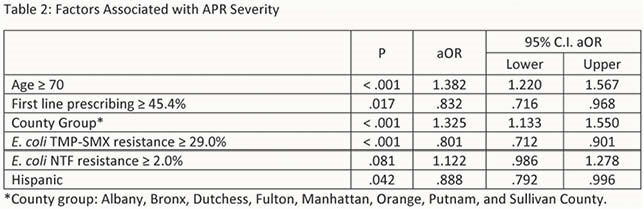

**Conclusion:**

Prescribing patterns may have a significant influence on initial presentation to the hospital for urinary tract infections. Outpatient antimicrobial stewardship should endeavor to promote guideline driven prescribing. Further research is needed to corroborate the findings from this cross-sectional study.

**Disclosures:**

**All Authors**: No reported disclosures

